# Gender violence and mental health in a series of cases

**DOI:** 10.1192/j.eurpsy.2026.11921

**Published:** 2026-07-31

**Authors:** M. Valverde Barea, M. D. R. Martos Castello, A. España Osuna, M. O. Solís Correa

**Affiliations:** 1 Psychiatry, H. U. Jaén, Jaén; 2 Psychiatry, H. Baza, Granada, Spain

## Abstract

**Introduction:**

Gender-based violence directly impacts the quality of life and mental health of the women who experience it. Violence against women is the leading cause of mental health problems, according to the World Health Organization. Women with dual pathologies are more susceptible to gender-based violence and suffer greater stigma and social exclusion. They also find it more difficult to access care services and therapeutic treatment.

**Objectives:**

Describe the interrelationship between gender violence and psychiatric pathology.

**Methods:**

We present a series of four clinical cases of women treated in the Short-Term Hospitalization Unit for acute psychiatric symptoms, manifesting as a suicidal attempt or psychotic episode. All of them were found to be actively using substances (alcohol, cocaine, or cannabis) upon admission, with a problematic pattern of use linked to past or present violence and used as a dysfunctional coping mechanism. In these contexts, intimate partner violence created an environment of heightened psychological vulnerability. The first case corresponds to a 31-year-old woman who presented an acute psychotic episode with delusional ideas of harm, suspicion, and self-referentiality, as well as delusional interpretations. The symptoms resolved with antipsychotic treatment along with cannabis abstinence. The remaining three cases correspond to women aged 54, 35, and 34, all of whom had used alcohol and/or cocaine. In these cases, the suicidal behavior materialized through the ingestion of psychotropic drugs, one of them in the context of acute intoxication. All patients received psychopharmacological and psychotherapeutic treatment during their stay and, after discharge, continued community follow-up in both mental health and addiction treatment and substance withdrawal.

**Results:**

The interrelationship between gender-based violence, substance use, and severe psychiatric symptoms poses a complex therapeutic approach and hinders treatment adherence.Early detection of these cases is essential to offer a comprehensive intervention that simultaneously addresses the psychiatric dimension, the violence experienced, and substance use.

**Conclusions:**

This series of cases highlights the need to strengthen interdisciplinary resources and support networks for women in this situation, given the high risk of chronicity and mortality associated with this clinical profile.

**Disclosure of Interest:**

None Declared

